# Clinical characteristics of asymptomatic carriers of novel coronavirus disease 2019: A multi-center study in Jiangsu Province

**DOI:** 10.1080/21505594.2020.1840122

**Published:** 2020-11-02

**Authors:** Jiaxin Chen, Tao Han, Mao Huang, Yi Yang, Futai Shang, Yishan Zheng, Wenjing Zhao, Liang Luo, Xudong Han, Aihua Lin, Hongsheng Zhao, Qin Gu, Yi Shi, Jun Li, Xingxiang Xu, Kexi Liu, Yijun Deng, Enzhi Jia, Quan Cao

**Affiliations:** a Department of Cardiovascular Medicine, The First Affiliated Hospital of Nanjing Medical University, Nanjing, Jiangsu Province, China; b Department of Critical Care Medicine, The First Affiliated Hospital of Nanjing Medical University, Nanjing, Jiangsu Province, China; c Department of Pulmonary and Critical Care Medicine, The First Affiliated Hospital of Nanjing Medical University, Nanjing, Jiangsu Province, China; d Department of Critical Care Medicine, Zhongda Hospital of Southeast University, Nanjing, Jiangsu Province, China; e Department of Critical Care Medicine, The Affiliated Huaian No. 1 People’s Hospital of Nanjing Medical University, Huaian, Jiangsu Province, China; f Department of Critical Care Medicine, Nanjing No.2 Hospital, Nanjing, Jiangsu Province, China; g Department of Critical Care Medicine, The Affiliated Hospital of Xuzhou Medical University, Xuzhou, Jiangsu Province, China; h Department of Critical Care Medicine, Wuxi No. 2 People's Hospital, Wuxi, Jiangsu Province, China; i Department of Critical Care Medicine, The Third People’s Hospital of Nantong City Affiliated to Nantong University, Nantong, Jiangsu Province, China; j Department of Critical Care Medicine, Suqian People’s Hospital of Nanjing Gulou Hospital Group, Suqian, Jiangsu Province, China; k Department of Critical Care Medicine, The Affiliated Hospital of Nantong University, Nantong, Jiangsu Province, China; l Department of Critical Care Medicine, Nanjing Drum Tower Hospital, The Affiliated Nanjing University Medical School, Nanjing, Jiangsu Province, China; m Department of Pulmonary and Critical Care Medicine, Nanjing Jinlin Hospital, Nanjing, Jiangsu Province, China; n Department of Infectious Diseases, The First Affiliated Hospital of Nanjing Medical University, Nanjing, Jiangsu Province, China; o Department of Pulmonary and Critical Care Medicine, Northern Jiangsu People’s Hospital, Yangzhou, Jiangsu Province, China; p Department of Critical Care Medicine, Lianyungang First People’s Hospital, Lianyungang, Jiangsu Province, China; q Department of Critical Care Medicine, Yancheng First People’s Hospital, Yancheng, Jiangsu Province, China

**Keywords:** Novel coronavirus disease-19, COVID-19, severe acute respiratory syndrome coronavirus 2, SARS-CoV-2, asymptomatic carriers, immune response, inflammatory marker, disease severity of COVID-19

## Abstract

Asymptomatic SARS-CoV-2-infected individuals are thought to play major roles in virus transmission. This study aimed to analyze the characteristics of asymptomatic carriers with COVID-19 to control the spread of the virus. We retrospectively investigated the clinical characteristics of 648 consecutive subjects who were enrolled in the study and were divided into asymptomatic carriers, mild cases, ordinary cases, severe or critical cases, and evaluated their impact on disease severity by means of Spearman correlation and multiple regression analyses. Receiver operating characteristic curve analysis was conducted to determine the optimum cutoff levels of laboratory findings for diagnostic predictors of asymptomatic carriers of COVID-19. In our study, a total of 648 subjects on admission with a mean age of 45.61 y including 345 males and 303 females were enrolled in our study. The leukocyte, lymphocyte, eosinophil, platelet, C-reactive protein, interleukin-6, CD3+, CD4+, and CD8 + T lymphocyte levels, and the erythrocyte sedimentation rate differed significantly among the groups (all *p* ≤ 0.05). Disease severity was negatively associated with the CD3+ (*r* = −0.340; *p* < 0.001), CD4+ (*r* = −0.290; *p* = 0.001) and CD8+ (*r* = −0.322; *p* < 0.001) T lymphocyte levels. The significant diagnostic predictors of asymptomatic carriers of COVID-19 included the blood cell, cytokine, and T lymphocyte subset levels. Inflammation and immune response may play important roles in disease progression. Hence, the laboratory parameters identified should be considered in clinical practice, which provide new insights into the identification of asymptomatic individuals and the prevention of virus transmission.

## Introduction

Novel coronavirus disease 2019 (COVID-19) is caused by infection with severe acute respiratory syndrome coronavirus 2 (SARS-CoV-2), and it has swept across 31 provinces in China [1] and over 200 countries worldwide [2]. The prevention and treatment of COVID-19 in China have achieved remarkable results, especially in Jiangsu Province. Since 14 March 2020, 631 patients with pneumonia caused by SARS-CoV-2 have recovered and been discharged from hospital in Jiangsu Province [3].

Genetic analyses of SARS-CoV-2 and severe acute respiratory syndrome coronavirus (SARS-CoV) have shown that the viruses share similar characteristics [4]. Previous studies’ findings have shown that a cytokine storm is one of the most important mechanisms underlying disease progression and death. Cytokine storms overstimulate the body’s immune response to microorganisms or drugs as a consequence of acute increases in the levels of inflammatory factors [5]. In the final stages of the disease, many patients with COVID-19 may develop acute respiratory distress syndrome or even multiple organ failure.

To prevent virus transmission, the characteristics of patients with asymptomatic SARS-CoV-2 infection have been studied. Evidence suggests that the SARS-CoV-2 loads are similar in asymptomatic and symptomatic patients, and that asymptomatic patients may continue to test positive for the virus for up to 21 d [6,7]. The report on the outbreak on the “Diamond Princess” cruise ship indicated that of 1723 travelers tested, 189 asymptomatic individuals tested positive for SARS-CoV-2, which suggested that many asymptomatic individuals remained undiscovered in the community [8]. Moreover, several disease clusters that included individuals who acquired SARS-CoV-2 from infected individuals and did not develop symptoms have been described [9–11]. Preventing transmission remains at the forefront of the current public health strategies for controlling the COVID-19 pandemic, but the presence of asymptomatic individuals poses huge challenges regarding the control of COVID-19 [12]. Accordingly, identifying and isolating individuals with asymptomatic COVID-19 are necessary to prevent subsequent outbreaks.

Therefore, the present study was conducted to investigate the clinical and laboratory characteristics of 648 subjects who comprised patients with asymptomatic COVID-19 and those with mild, ordinary, and severe or critical disease, and to compare the four groups in relation to the patients’ immune responses to infection, which involved evaluating the markers of inflammation and T lymphocyte subsets. In addition, we aimed to explore potential diagnostic predictors of asymptomatic SARS-CoV-2 infection that may help to identify and screen these patients.

## Patients and methods

This study was approved by the ethics committee of Nanjing Medical University and the First Affiliated Hospital of Nanjing Medical University, Jiangsu Province, China, and it conformed to the ethical principles of the Declaration of Helsinki. The need for written informed consent was waived, because of the study’s retrospective design and the urgent need to collect and analyze data. The authors were members of the Novel Coronavirus Pneumonia Prevention and Control Group in Jiangsu Province, and they were supported by the Jiangsu Provincial Commission of Health. The study was designed by the investigators under the supervision of the Jiangsu Provincial Commission of Health.

### Study participants

From 23 January 2020 to 11 March 2020, 648 consecutive subjects, comprising 345 males and 303 female patients, who were aged from 1 y to 98 y and were from 25 hospitals in Jiangsu Province, China, were enrolled in this study. The patients’ epidemiological data, demographic data, clinical characteristics, radiographic characteristics, and key laboratory parameters were analyzed.

The *Guidance for Corona Virus Disease 2019: Prevention, Control, Diagnosis and Management* (sixth edition), which was issued by China’s National Health Commission, was used to classify the patients with mild, ordinary, severe, and critical disease according to the severity of the COVID-19 symptoms. Patients with (1) mild disease present with mild symptoms only without radiographic features; (2) ordinary disease present with fever, respiratory symptoms, and radiographic features; (3) severe disease meet one of the following three criteria, namely, dyspnea, which is defined as a respiratory rate >30 times/min, an oxygen saturation of <93% in ambient air, or a ratio of arterial oxygen partial pressure to fractional inspired oxygen <300 mmHg; and (4) critical disease meet one of the following three criteria, namely, respiratory failure, septic shock, or multiple organ failure [13].

Individuals with asymptomatic COVID-19 were laboratory-confirmed as positive for SARS-CoV-2 by testing pharyngeal or anal swab samples for SARS-CoV-2 nucleic acids; these individuals did not show any obvious symptoms during nucleic acid screening [13]. The asymptomatic individuals were identified mainly by investigating clusters of outbreaks and tracking infectious individuals whose computed tomography (CT) images were normal and who had no symptoms on admission to hospital or during hospitalization.

We combined the patients with severe or critical disease into one group for further analysis, because of the small numbers of patients present in each group. Therefore, our study comprised four groups, namely, 50 asymptomatic carriers, 81 mild cases, 486 ordinary cases, and 31 severe or critical cases of COVID-19.

### Data collection

We obtained the medical records and compiled data for hospitalized patients with laboratory-confirmed COVID-19 from 25 hospitals in Jiangsu Province ranging from 23 January 2020 to 11 March 2020. Then, we extracted data from the medical records that described the patients’ recent exposure histories, clinical symptoms or signs, and laboratory findings on admission. The radiologic assessments included chest radiography or CT scans, and all laboratory testing was performed according to the patients’ clinical care needs. The laboratory tests investigated the patients’ complete blood counts, blood biochemistry, coagulation parameters, inflammatory marker levels, namely, the C-reactive protein (CRP), procalcitonin (PCT), and interleukin (IL)-6 levels, erythrocyte sedimentation rates (ESRs), and lymphocyte subset levels, that is, cluster of differentiation (CD)3+, CD4+, and CD8 + T lymphocytes.

### Statistical analyses

Data were analyzed using the Statistics Package for Social Sciences (ver. 16.0; SPSS Incorporated, Chicago, IL, USA). Normally distributed variables were presented as mean ± standard deviation (SD), and the comparisons were analyzed using Analysis of variance (ANOVA). Otherwise, variables with a skewed distribution were presented as median and quartile ranges, and the comparisons were made using the Kruskal–Wallis H test. Categorical variables were compared using Chi-Square analyses. Moreover, disease severity was scored according to the *Guidance for Corona Virus Disease 2019: Prevention, Control, Diagnosis and Management* (sixth edition), which was issued by China’s National Health Commission [13]. Asymptomatic carriers with COVID-19 were scored with 1 while mild cases, ordinary cases, severe or critical cases were scored with 2, 3, 4, respectively. Spearman two-way test and multiple regression analysis were used to assess the relationship between disease severity of COVID-19 and laboratory characteristics. Furthermore, we redefined asymptomatic carriers with COVID-19 as group 1, while the other symptomatic infections were group 0. Then, the receiver operating characteristic (ROC) curve analysis was conducted to determine the optimum cutoff levels of laboratory findings with the sensitivity, the specificity and Youden index for diagnostic predictor of asymptomatic carriers with COVID-19. Two-tailed *P* value less than 0.05 was considered statistically significant.

## Results

### Demographics, and the baseline and clinical characteristics of the study subjects according to disease severity of COVID-19

In the present study, 50 asymptomatic carriers, 81 mild cases, 486 ordinary cases, 31 severe or critical cases with COVID-19 from 25 hospitals in Jiangsu Province were investigated. Demographics, the baseline and clinical characteristics of 648 subjects infected with SARS-CoV-2 are presented in Table 1.

**Table 1.** Clinical characteristics of the study subjects according to the disease severity of COVID-19

Characteristic	Disease severity of COVID-19				Statistic parameter	
	Asymptomatic carriers (n = 50)	Mild cases (n = 81)	Ordinary cases (n = 486)	Severe or critical cases (n = 31)	*F* value/*Chi-Square*	*P* value
Mean (SD) age, years	41.37 ± 23.96 (n = 49)	39.85 ± 18.08 (n = 81)	46.03 ± 16.12 (n = 486)	60.84 ± 13.55 (n = 31)	12.543	< 0.001
Sex, male, n (%)	23/50 (46.0)	35/81 (43.2)	267/486 (54.9)	20/31 (64.5)	6.472	0.091
Smoker, n (%)	6/50 (12.0)	3/81 (3.7)	40/486 (8.2)	2/31 (6.5)	3.288	0.349
Exposure to source of transmission within 14 d before admission, n/total n (%)						
Living in Wuhan	3/50 (6.0)	6/81 (7.4)	62/486 (12.8)	1/31 (3.2)	5.611	0.132
Contact with wildlife	0/50 (0)	0/81 (0)	1/486 (0.2)	0/31 (0)	0.334	0.954
Visited Wuhan recently	3/50 (6.0)	16/81(19.8)	90/486 (18.5)	4/31 (12.9)	5.682	0.128
Contact with Wuhan residents	14/50 (28.0)	14/81 (17.3)	121/486 (24.9)	4/31 (12.9)	4.748	0.191
Temperature on admission (°C)	36.65(36.40–36.80) (n = 48)	36.80 (36.53–37.28) (n = 80)	36.90 (36.50–37.50) (n = 475)	36.65 (36.50–37.20) (n = 30)	18.857	< 0.001
Fever	0/50 (0)	41/81 (50.6)	382/486 (78.6)	28/31 (90.3)	153.20	< 0.001
Symptoms on admission, n (%)						
Conjunctival congestion	0/50 (0)	1/81 (1.2)	1/486 (0.2)	0/31 (0)	2.675	0.445
Nasal congestion	0/50 (0)	21/81 (25.9)	30/486 (6.2)	0/31 (0)	45.269	< 0.001
Headache	0/50 (0)	4/81 (4.9)	52/486 (10.7)	1/31 (3.2)	9.719	0.021
Cough	0/50 (0)	29/81 (35.8)	327/486 (67.3)	23/31 (74.2)	106.30	< 0.001
Sore throat	0/50 (0)	8/81 (9.9)	57/486 (11.7)	6/31 (19.4)	8.787	0.032
Sputum production	0/50 (0)	15/81 (18.5)	185/486 (38.1)	14/31 (45.2)	40.009	< 0.001
Fatigue	0/50 (0)	6/81 (7.4)	154/486 (31.7)	12/31 (38.7)	42.229	< 0.001
Hemoptysis	0/50 (0)	1/81 (1.2)	6/486 (1.2)	0/31 (0)	1.011	0.799
Shortness of breath	0/50 (0)	2/81 (2.5)	96/486 (19.8)	22/31 (71.0)	82.198	< 0.001
Nausea or vomiting	0/50 (0)	1/81 (1.2)	31/486 (6.4)	3/31 (9.7)	7.652	0.054
Diarrhea	0/50 (0)	5/81 (6.2)	56/486 (11.5)	8/31 (25.8)	15.541	0.001
Myalgia or arthralgia	0/50 (0)	9/81 (11.1)	83/486 (17.1)	4/31 (12.9)	11.639	0.009
Chills	0/50 (0)	2/81 (2.5)	65/486 (13.4)	6/31 (19.4)	16.809	0.001
Signs of infection on admission, n (%)						
Throat congestion	0/50 (0)	17/81 (21.0)	60/486 (12.3)	2/31 (6.5)	14.102	0.029
Swollen tonsils	0/50 (0)	0/81 (0)	2/486 (0.4)	0/31 (0)	0.669	0.881
Lymph-node enlargement	0/50 (0)	0/81 (0)	0/486 (0)	0/31 (0)	NA	NA
Rash	0/50 (0)	0/81 (0)	1/486 (0.1)	1/31 (3.2)	9.147	0.027

SD: standard deviation; NA: not applicable.

The data presented are the means (standard deviations), numbers (percentages), or the medians and the interquartile ranges. The denominators of the patients included in the analysis are provided if they differed from the overall numbers in the group. Rounding may mean the percentages do not total 100.

None of the asymptomatic patients who were infected with SARS-CoV-2 had symptoms on admission to hospital or during hospitalization. The four patient groups differed significantly regarding age (*p* < 0.001), the temperature on admission (*p* < 0.001), fever (*p* < 0.001), nasal congestion (*p* < 0.001), headache (*p* = 0.021), cough (*p* < 0.001), sore throat (*p* = 0.032), sputum production (*p* < 0.001), fatigue (*p* < 0.001), shortness of breath (*p* < 0.001), myalgia or arthralgia (*p* = 0.009), chills (*p* = 0.001), and throat congestion (*p* = 0.029). The groups did not differ with regard to sex, the cigarette smoking status, the epidemiological history, conjunctival congestion, hemoptysis, and swollen tonsils.

### Chest imaging results of the study subjects according to disease severity of COVID-19

Table 2 presents the chest imaging results from the patients on admission to hospital. Of the 648 subjects, 354 subjects’ chest imaging results were analyzed; the remaining patients’ imaging data were missing. Chest imaging showed ground-glass opacity (GGO) (146/354), local patchy shadowing (69/354), bilateral patchy shadowing (152/354), and interstitial abnormalities (13/354). Significant differences were evident among the groups in relation to GGO (*p* = 0.022) and bilateral patchy shadowing (*p* < 0.001).

**Table 2.** Radiographic characteristics of the study subjects according to the disease severity of COVID-19

Characteristic	Disease severity of COVID-19				Statistic parameter	
	Asymptomatic carriers (n = 50)	Mild cases (n = 81)	Ordinary cases (n = 486)	Severe or critical cases (n = 31)	*Chi-Square*	*P* value
Abnormalities on chest computed tomography images before admission n/total n (%)						
Ground-glass opacity	2/14 (14.3)	3/18 (16.7)	133/302 (44.0)	8/20 (40.0)	9.672	0.022
Local patchy shadowing	3/14 (21.4)	2/18 (11.1)	63/302 (20.9)	1/20 (5.0)	3.876	0.275
Bilateral patchy shadowing	3/14 (21.4)	1/18 (5.6)	133/302 (44.0)	15/20 (75.0)	21.451	< 0.001
Interstitial abnormalities	1/14 (7.1)	0/18 (0)	12/302 (4.0)	0/20 (0)	2.003	0.572

The data presented are numbers (percentages).

### Laboratory measurements of the study subjects according to disease severity of COVID-19

Table 3 shows the laboratory test results from the study subjects stratified according to the severity of the disease on admission to hospital. The groups differed significantly in relation to the leukocyte (*p* < 0.001), lymphocyte (*p* < 0.001), eosinophil (*p* < 0.001), platelet (*p* < 0.001), and neutrophil (*p* = 0.003) counts. The median lymphocyte counts were 1.74 (IQR, 1.37–2.79) for the asymptomatic individuals and 0.64 (IQR, 0.46–1.03) for the 31 severe or critical cases. Regarding liver and renal function, significant differences were evident among the groups in relation to the creatinine (Cr) (*p* = 0.013), blood urea nitrogen (*p* = 0.002), albumin (*p* < 0.001), aspartate aminotransferase (AST) (*p* < 0.001), alkaline phosphatase (ALP) (*p* = 0.001), lactate dehydrogenase (LDH) (*p* = 0.001), potassium (*p* = 0.014), and sodium (*p* = 0.009) levels. The total serum bilirubin (*p* = 0.100) and alanine aminotransferase (*p* = 0.107) levels did not differ among the groups. The prolonged prothrombin time (*p* = 0.012), activated partial thromboplastin time (APTT) (*p* = 0.014), and fibrinogen levels (*p* < 0.001) differed according to disease severity.

**Table 3.** Laboratory measurements of the study subjects according to the disease severity of COVID-19

Characteristic	Disease severity of COVID-19				Statistic parameter	
	Asymptomatic carriers (n = 50)	Mild cases (n = 81)	Ordinary cases (n = 486)	Severe or critical cases (n = 31)	*F* value/ *Chi-Square*	*P* value
Routine blood test						
Hemoglobin (g/L)	141.00 (127.25–153.75) (n = 36)	135.00 (123.00–150.00) (n = 57)	140.00 (128.00–152.00) (n = 318)	129.50 (121.25–156.75) (n = 24)	3.379	0.337
Platelet count (× 10^9^/L)	230.50(191.00–255.00) (n = 40)	209.00 (158.75–269.50) (n = 62)	178.00 (142.00–218.00) (n = 349)	141.00 (116.00–168.00) (n = 23)	40.939	< 0.001
White blood cell count (× 10^9^/L)	6.35 (5.03–7.38) (n = 40)	5.44 (4.63–6.97) (n = 66)	4.65 (3.80–5.83) (n = 373)	4.58 (4.00–7.47) (n = 24)	31.543	< 0.001
Neutrophil count (× 10^9^/L)	3.46 (2.40–4.52) (n = 38)	3.23 (2.48–4.57) (n = 62)	2.79 (2.09–3.87) (n = 367)	3.93 (2.71–6.76) (n = 23)	13.626	0.003
Monocyte count (× 10^9^/L)	0.50 ± 0.23 (n = 11)	0.44 ± 0.16 (n = 12)	0.41 ± 0.18 (n = 109)	0.33 ± 0.18 (n = 13)	1.928	0.128
Eosinophil count (× 10^9^/L)	0.04 (0.02–0.14) (n = 40)	0.03 (0.01–0.07) (n = 67)	0.01 (0.00–0.04) (n = 377)	0.01 (0.00–0.01) (n = 23)	35.756	< 0.001
Lymphocyte count (× 10^9^/L)	1.74 (1.37–2.79) (n = 38)	1.64 (1.05–2.20) (n = 62)	1.18 (0.87–1.59) (n = 367)	0.64 (0.46–1.03) (n = 24)	64.581	< 0.001
Blood biochemistry indicators						
Potassium (mmol/L)	3.98 (3.51–4.32) (n = 32)	3.83 (3.55–4.19) (n = 57)	3.80 (3.58–4.12) (n = 311)	3.65 (3.35–3.75) (n = 18)	10.543	0.014
Sodium (mmol/L)	139.80 (137.85–141.78) (n = 32)	138.90 (136.35–141.40) (n = 57)	138.20 (136.00–140.78) (n = 312)	135.38 (133.00–139.10) (n = 18)	11.626	0.009
Blood urea nitrogen (mmol/L)	4.24 (3.40–5.11) (n = 40)	3.90 (3.00–4.52) (n = 58)	3.80 (3.08–4.68) (n = 324)	5.29 (4.19–12.41) (n = 17)	14.798	0.002
Creatinine (µmol/L)	56.50 (45.00–75.20) (n = 40)	53.00 (44.40–63.00) (n = 59)	62.20 (50.00–75.00) (n = 361)	67.00 (32.00–78.75) (n = 21)	10.726	0.013
Total serum bilirubin (µmol/L)	10.50 (7.46–15.75) (n = 38)	8.50 (6.80–14.40) (n = 65)	10.90 (7.50–15.38) (n = 364)	13.20 (8.69–16.50) (n = 20)	6.255	0.100
Albumin (g/L)	45.15 (43.00–48.53) (n = 38)	44.40 (41.55–48.40) (n = 65)	42.30 (39.10–45.50) (n = 342)	39.90 (32.75–44.25) (n = 17)	29.493	< 0.001
Aspartate aminotransferase (U/L) (n = 390)	20.70 (16.60–23.00) (n = 27)	21.00 (16.60–27.00) (n = 47)	25.00 (19.40–33.40) (n = 297)	43.00 (28.00–50.00) (n = 19)	33.585	< 0.001
Alanine aminotransferase (U/L) (n = 464)	22.00 (16.50–31.70) (n = 37)	23.20 (16.00–31.50) (n = 63)	25.00 (17.30–38.00) (n = 347)	30.80 (25.10–50.60) (n = 17)	6.094	0.107
Alkaline phosphatase (U/L)	84.10 (63.00–107.50) (n = 37)	70.00 (51.00–90.50) (n = 65)	65.00 (53.00–79.00) (n = 346)	67.00 (59.25–73.95) (n = 16)	15.747	0.001
Lactate dehydrogenase (U/L)	209.00(172.50–277.75) (n = 36)	194.00 (164.00–314.00) (n = 61)	228.00 (181.00–317.50) (n = 340)	375.00 (248.00–458.50) (n = 17)	16.800	0.001
Hemostasis parameters						
Prothrombin time (s)	12.00 (11.28–12.60) (n = 42)	11.90 (11.20–12.70) (n = 71)	12.20 (11.38–13.10) (n = 422)	12.80 (11.20–13.70) (n = 27)	10.980	0.012
Activated partial thromboplastin time (s)	29.90 (26.25–35.80) (n = 41)	29.60 (26.50–36.05) (n = 69)	31.85 (28.70–36.90) (n = 422)	33.60 (31.80–36.80) (n = 27)	10.552	0.014
Fibrinogen (g/L)	2.64 (2.17–3.03) (n = 38)	3.01(2.57–3.55) (n = 64)	3.62 (2.86–4.33) (n = 401)	4.53 (2.84–6.07) (n = 26)	47.472	< 0.001

### Levels of Inflammatory markers and lymphocyte subsets of the study subjects according to disease severity of COVID-19

Table 4 presents the results from the inflammatory marker and lymphocyte subset assays of the blood from the subjects infected with SARS-CoV-2. Statistical differences were evident among the four groups regarding the ESR (*p* < 0.001), CRP level (*p* < 0.001), and PCT level (*p* = 0.004). Lymphocyte subset analyses revealed that the numbers of CD3+, CD4+, and CD8 + T lymphocytes in the patients with mild, ordinary, and severe or critical COVID-19 were significantly lower than those in the patients with asymptomatic disease (*p* = 0.001, *p* = 0.012, and *p* = 0.001, respectively).

**Table 4.** Levels of Inflammatory markers and lymphocyte subsets of the study subjects according to disease severity of COVID-19

Characteristic	Disease severity of COVID-19					Statistic parameter	
	Asymptomatic carriers (n = 50)	Mild cases (n = 81)	Ordinary cases (n = 486)	Severe or critical cases (n = 31)		*F* value/*Chi-Square*	*P* value
Inflammatory marker							
Erythrocyte sedimentation rate (mm/h)	7.00 (4.25–13.00) (n = 32)	11.00 (5.00–25.75) (n = 38)	18.00 (8.00–32.00) (n = 239)		58.00 (33.50–75.00) (n = 17)	37.498	< 0.001
C-reactive protein > 10.0 mg/L	7/39 (17.9)	12/62 (19.4)	173/359 (48.2)		23/26 (88.5)	49.374	< 0.001
Procalcitonin (ng/mL)	0.03 (0.02–0.10) (n = 23)	0.03 (0.02–0.05) (n = 38)	0.04 (0.02–0.08) (n = 241)		0.15 (0.06–0.28) (n = 14)	13.243	0.004
Interleukin-6 (pg/mL)	0.01 (0.01–0.02) (n = 11)	0.01 (0.01–2.32) (n = 21)	0.02 (0.01–0.04) (n = 92)		0.03 (0.03–0.15) (n = 5)	5.774	0.123
Lymphocyte subset							
CD3 + T lymphocytes (/µL)	1411.54 ± 671.25 (n = 13)	1431.06 ± 577.90 (n = 16)	1045.06 ± 501.65 (n = 89)		486.60 ± 266.28 (n = 5)	6.219	0.001
CD4 + T lymphocytes (/µL)	782.69 ± 366.39 (n = 13)	712.56 ± 350.52 (n = 16)	577.25 ± 300.94 (n = 89)		307.80 ± 164.67 (n = 5)	3.803	0.012
CD8 + T lymphocytes (/µL)	451.00 (286.50–634.00) (n = 13)	532.00(329.25–663.50) (n = 16)	316.00(216.00–526.50) (n = 89)		144.00 (62.50–259.00) (n = 5)	15.533	0.001

CD: cluster of differentiation.

### Spearman correlations between disease severity of COVID-19 and age, laboratory parameters

Correlations between disease severity and age, laboratory parameters were assessed using Spearman correlation coefficient (Table 5). Disease severity was positively associated with age (*r* = 0.178; *p* < 0.001), the Cr (*r* = 0.121; *p* = 0.008), AST (*r* = 0.286; *p* < 0.001), LDH (*r* = 0.159; *p* = 0.001), fibrinogen (*r* = 0.294; *p* < 0.001), PCT (*r* = 0.165; *p* = 0.003), and IL-6 (*r* = 0.183; *p* = 0.038) levels, the PT (*r* = 0.135; *p* = 0.001), the APTT (*r* = 0.137; *p* = 0.001), and the ESR (*r* = 0.311; *p* < 0.001), and negatively associated with the leukocyte (*r* = −0.214; *p* < 0.001), lymphocyte (*r* = −0.357; *p* < 0.001), eosinophil (*r* = −0.259; *p* < 0.001), platelet (*r* = −0.289; *p* < 0.001), sodium (*r* = −0.157; *p* = 0.001), potassium (*r* = −0.110; *p* = 0.025), albumin (*r* = −0.251; *p* < 0.001), ALP (*r* = −0.147; *p* = 0.001), CD3 + T lymphocyte (*r* = −0.340; *p* < 0.001), CD4 + T lymphocyte (*r* = −0.290; *p* = 0.001), and CD8 + T lymphocyte (*r* = −0.322; *p* < 0.001) levels.

**Table 5.** Spearman correlations between disease severity of COVID-19 and age, laboratory characteristics

Variables	Disease severity of COVID-19	
	Correlation coefficient	*P* value
Age (years) (n = 647)	0.178	< 0.001
White blood cell count (×10^9^/L) (n = 503)	−0.214	< 0.001
Neutrophils count (×10^9^/L) (n = 490)	−0.051	0.257
Eosinophils count (×10^9^/L) (n = 507)	−0.259	< 0.001
Lymphocyte count (×10^9^/L) (n = 491)	−0.357	< 0.001
Platelet count (×10^9^/L) (n = 474)	−0.289	< 0.001
Monocytes count (×10^9^/L) (n = 145)	−0.189	0.023
Hemoglobin (g/L) (n = 435)	0.002	0.961
Potassium (mmol/L) (n = 418)	−0.110	0.025
Sodium (mmol/L) (n = 419)	−0.157	0.001
Blood Urea nitrogen (mmol/L) (n = 439)	−0.040	0.409
Creatinine (umol/L) (n = 481)	0.121	0.008
Total serum bilirubin (umol/L) (n = 487)	0.090	0.046
Albumin (g/L) (n = 462)	−0.251	< 0.001
Aspartate aminotransferase (U/L) (n = 390)	0.286	< 0.001
Alanine aminotransferase (U/L) (n = 464)	0.107	0.021
Alkaline phosphatase (U/L) (n = 464)	−0.147	0.001
Lactate dehydrogenase (U/L) (n = 454)	0.159	0.001
Prothrombin time (s) (n = 562)	0.135	0.001
Activated partial thromboplastin time (s) (n = 559)	0.137	0.001
Fibrinogen (g/L) (n = 529)	0.294	< 0.001
Erythrocyte sedimentation rate (mm/h) (n = 326)	0.311	< 0.001
Procalcitonin (ng/mL) (n = 316)	0.165	0.003
Interleukin-6 (pg/ml) (n = 129)	0.183	0.038
CD3 + T lymphocytes (/ul) (n = 123)	−0.340	< 0.001
CD4 + T lymphocytes (/ul) (n = 123)	−0.290	0.001
CD8 + T lymphocytes (/ul) (n = 123)	−0.322	< 0.001

CD: cluster of differentiation.

### Association between disease severity of COVID-19 and laboratory characteristics by multiple regression analysis

Multiple regression analysis was performed to investigate any independent association among four groups according to disease severity of COVID-19 (Table 6). In comparison with the asymptomatic carriers (group 1), the mild (group 2), ordinary (group 3), severe or critical (group 4) cases were independently associated with the level of erythrocyte sedimentation rate (ESR), with ORs of 1.044 (95% *CI*, 1.005‐1.085), 1.047 (95% *CI*, 1.011‐1.084) and 1.086 (95% *CI*,1.044‐1.129), respectively, after adjustment for the factor of age. Compared to group 1, the ordinary cases, severe or critical cases were associated with the level of lymphocytes, with ORs of 0.383 (95% *CI*, 0.259–0.567) and 0.029 (95% *CI*, 0.007–0.114); platelet, with ORs of 0.991 (95% *CI*, 0.986–0.995) and 0.979 (95% *CI*, 0.969–0.989); albumin, with ORs of 0.881 (95% *CI*, 0.820–0.947) and 0.837 (95% *CI*, 0.754–0.930); fibrinogen, with ORs of 2.514 (95% *CI*, 1.661–3.888) and 3.597 (95% *CI*, 2.171–5.960); C-reactive protein, with ORs of 0.246 (95% *CI*, 0.103–0.585) and 0.042 (95% *CI*, 0.010–0.186); CD3 + T lymphocyte, with ORs of 0.999 (95% *CI*, 0.998–1.000) and 0.995 (95% *CI*, 0.990–0.999); CD4 + T lymphocyte, with ORs of 0.998 (95% *CI*, 0.997–1.000) and 0.994 (95% *CI*, 0.987–1.000).

**Table 6.** Association between disease severity of COVID-19 and laboratory characteristics by multiple regression analysis

Variable	Mild cases (n = 81)		Ordinary cases (n = 486)		Severe and critical cases (n = 31)	
	OR(95%*CI*)	*P* value	OR(95%*CI*)	*P* value	OR(95%*CI*)	*P* value
Platelet count (×10^9^/L) (n = 474)	0.997(0.992–1.002)	0.199	0.991(0.986–0.995)	< 0.001	0.979(0.969–0.989)	< 0.001
White blood cell count (×10^9^/L) (n = 503)	0.923(0.779–1.093)	0.351	0.755(0.649–0.879)	< 0.001	0.913(0.725–1.149)	0.437
Lymphocyte count (×10^9^/L) (n = 491)	0.794(0.564–1.118)	0.187	0.383(0.259–0.567)	< 0.001	0.029(0.007–0.114)	< 0.001
Potassium (mmol/L) (n = 418)	0.685(0.351–1.336)	0.267	1.010(0.836–1.221)	0.917	0.283(0.097–0.830)	0.021
Albumin (g/L) (n = 462)	0.953(0.877–1.036)	0.258	0.881(0.820–0.947)	0.001	0.837(0.754–0.930)	0.001
Aspartate aminotransferase (U/L) (n = 390)	0.987(0.933–1.045)	0.657	1.045(1.000–1.093)	0.052	1.071(1.021–1.124)	0.005
Alanine aminotransferase (U/L) (n = 464)	1.008(0.983–1.034)	0.517	1.018(0.996–1.040)	0.109	1.031(1.004–1.058)	0.024
Fibrinogen (g/L) (n = 529)	1.512(0.929–2.460)	0.096	2.514(1.661–3.888)	< 0.001	3.597(2.171–5.960)	< 0.001
Erythrocyte sedimentation rate (mm/h) (n = 326)	1.044(1.005–1.085)	0.028	1.047(1.011–1.084)	0.009	1.086(1.044–1.129)	< 0.001
C-reactive protein (mg/L) (n = 486)	0.882(0.305–2.549)	0.817	0.246(0.103–0.585)	0.002	0.042(0.010–0.186)	< 0.001
CD3 + T lymphocytes (/ul) (n = 123)	1.000(0.999–1.001)	0.777	0.999(0.998–1.000)	0.040	0.995(0.990–0.999)	0.024
CD4 + T lymphocytes (/ul) (n = 123)	0.999(0.997–1.001)	0.382	0.998(0.997–1.000)	0.050	0.994(0.987–1.000)	0.042
CD8 + T lymphocytes (/ul) (n = 123)	1.000(0.998–1.003)	0.717	0.999(0.996–1.001)	0.215	0.998(0.976–1.000)	0.047

CD: cluster of differentiation; OR: odds ratio; CI: confidence interval.

### Receiver operating characteristic curve analyses for diagnostic predictor of asymptomatic carriers with COVID-19

**Figure 1.**
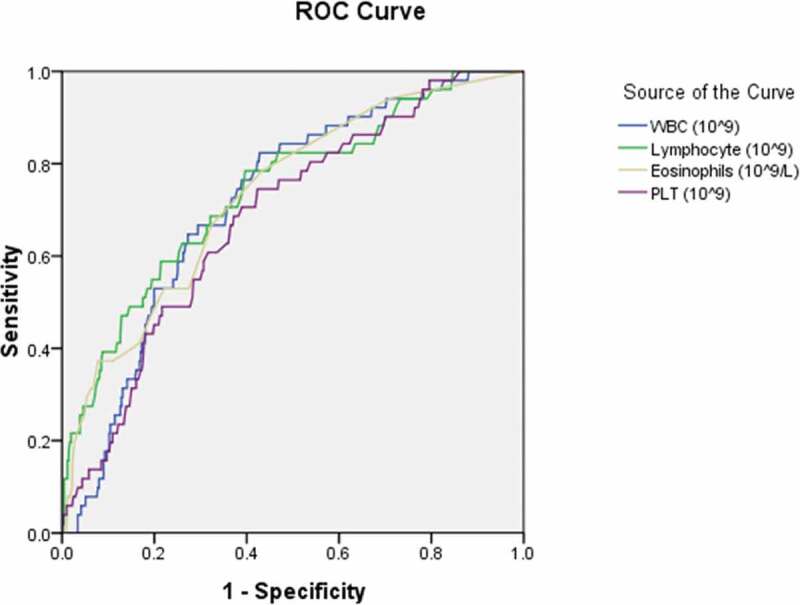
Receiver operating characteristic curve analyses of leukocytes, lymphocytes, eosinophils, and platelets as diagnostic predictors of asymptomatic carriers of COVID-19. ROC: receiver operating characteristic; WBC: white blood cell; PLT: platelet

**Figure 2.**
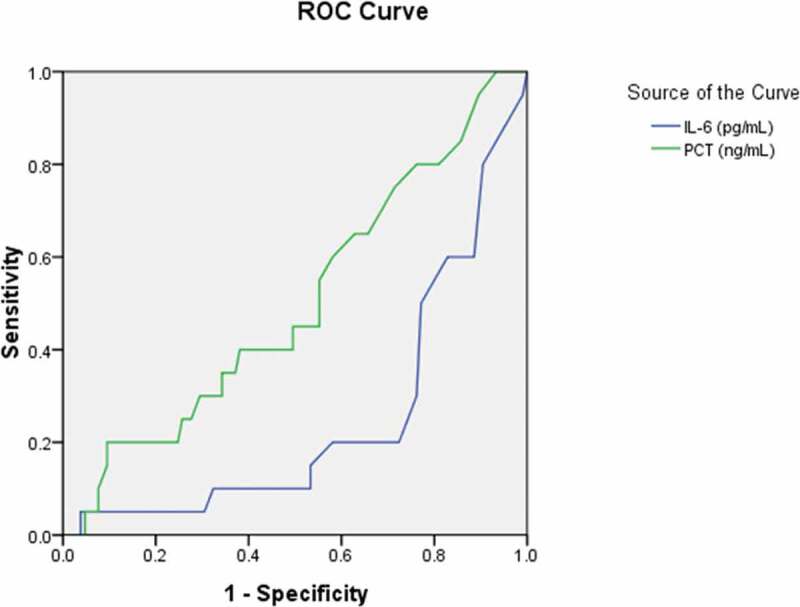
Receiver operating characteristic curve analyses of interleukin-6 and procalcitonin as diagnostic predictors of asymptomatic asymptomatic carriers of COVID-19. ROC: receiver operating characteristic; IL-6: interleukin-6; PCT: procalcitonin

**Figure 3.**
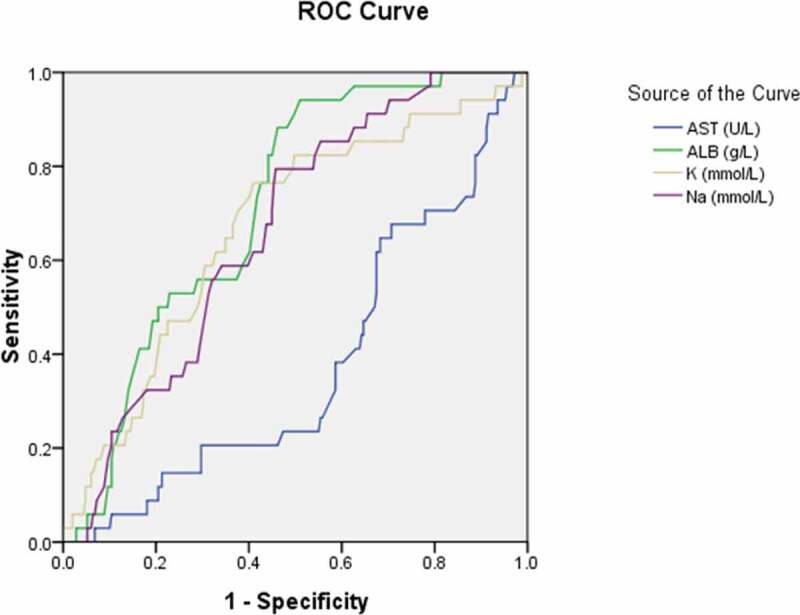
Receiver operating characteristic curve analyses of aspartate aminotransferase, albumin, potassium, and sodium as diagnostic predictors of asymptomatic asymptomatic carriers of COVID-19. ROC: receiver operating characteristic; AST: aspartate aminotransferase; ALB: albumin; K: potassium; Na: sodium

**Figure 4.**
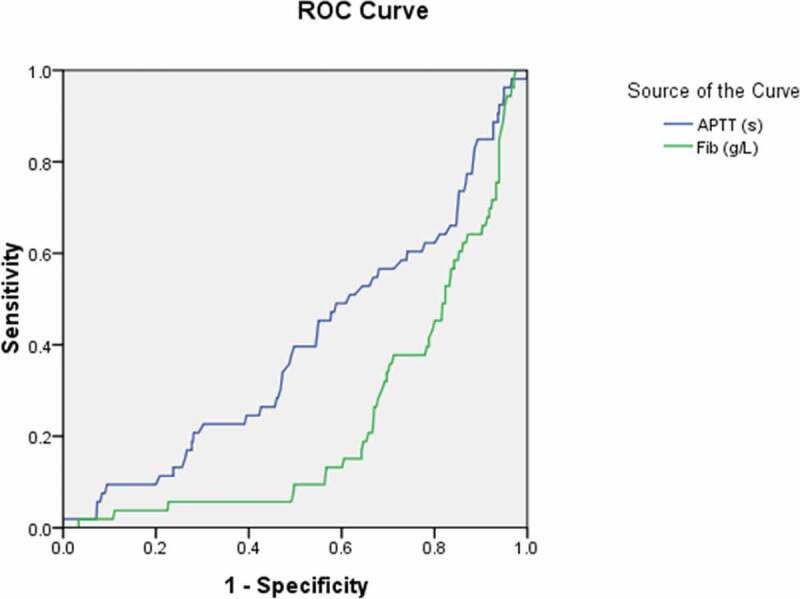
Receiver operating characteristic curve analyses of the activated partial thromboplastin time and fibrinogen as diagnostic predictors of asymptomatic asymptomatic carriers of COVID-19. ROC: receiver operating characteristic; APTT: activated partial thromboplastin time; FIB: fibrinogen

**Figure 5.**
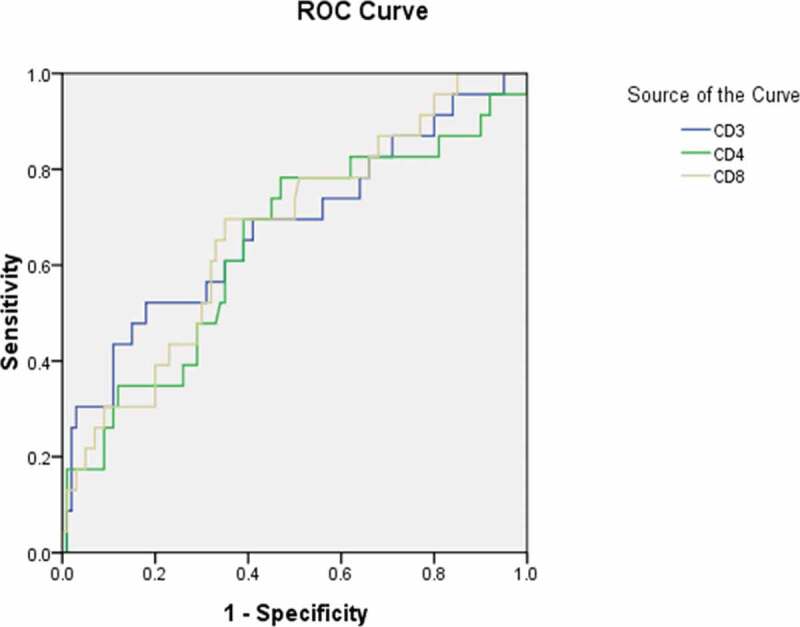
Receiver operating characteristic curve analyses of cluster of differentiation (CD)3+, CD4+, and CD8+ lymphocyte levels as diagnostic predictors of asymptomatic asymptomatic carriers of COVID-19. ROC: receiver operating characteristic; CD: cluster of differentiation

To further explore the diagnostic predictor of asymptomatic carriers with COVID-19, subsequent ROC analyses were performed. According to You-den index and the receiver operating characteristic (ROC) curve, the best diagnostic cutoff value of variables for asymptomatic carriers with COVID-19 was determined with the sensitivity and specificity (Table 7; Figures 1,2,3,4 and 5). There were significant differences in the diagnostic cutoff value of leukocyte (95% *CI*: 0.647–0.782, *p* < 0.001), lymphocyte (95% *CI*: 0.665–0.814, *p* < 0.001), eosinophils (95% *CI*: 0.651–0.789, *p* < 0.001), platelet (95% *CI*: 0.626–0.766, *p* = 0.036) for identifying asymptomatic carriers of COVID-19 (Figure 1).

**Table 7.** Receiver operating characteristic curve analyses for diagnostic predictor of asymptomatic carriers of COVID-19

Variables	AUC (95% CI)	*P* value	Cutoff	Sensitivity	Specificity	Youden index
White blood cell count (×10^9^/L) (n = 503)	0.715(0.647–0.782)	< 0.001	5.015	0.811	0.571	0.382
Lymphocyte count (× 10^9^/L) (n = 491)	0.740(0.665–0.814)	< 0.001	1.365	0.784	0.598	0.382
Eosinophils count (×10^9^/L) (n = 507)	0.720(0.651–0.789)	< 0.001	0.015	0.792	0.548	0.340
Neutrophils count(×10^9^/L) (n = 490)	0.585(0.506–0.663)	0.048	3.5	0.549	0.654	0.203
Platelet count (×10^9^/L) (n = 474)	0.696(0.626–0.766)	0.036	190.5	0.755	0.582	0.337
Hemoglobin (g/L) (n = 435)	0.520(0.434–0.607)	0.651	121.5	0.894	0.178	0.072
Monocytes count (×10^9^/L) (n = 145)	0.632(0.473–0.790)	0.147	0.365	0.818	0.5	0.318
Potassium (mmol/L) (n = 418)	0.632(0.545–0.720)	0.003	3.885	0.681	0.604	0.285
Sodium (mmol/L) (n = 419)	0.648(0.579–0.718)	0.001	139.17	0.681	0.594	0.275
Blood Urea nitrogen (mmol/L) (n = 439)	0.574(0.498–0.651)	0.077	3.995	0.648	0.561	0.209
Creatinine (umol/L) (n = 481)	0.425(0.339–0.512)	0.074	15.1	0.981	0.047	0.028
Aspartate aminotransferase (U/L) (n = 390)	0.358(0.271–0.445)	0.004	13.55	1	0.028	0.028
Total serum bilirubin(umol/L) (n = 487)	0.481(0.400–0.562)	0.658	5.95	0.904	0.136	0.04
Alanine-aminotransferase (U/L) (n = 464)	0.449(0.367–0.532)	0.237	6.40	1	0.007	0.007
Alkaline phosphatase (U/L) (n = 464)	0.695(0.611–0.778)	< 0.001	82.70	0.569	0.797	0.366
Albumin (g/L) (n = 462)	0.692(0.627–0.758)	< 0.001	42.65	0.846	0.52	0.366
Lactate dehydrogenase (U/L) (n = 454)	0.425(0.348–0.501)	0.082	142.00	1	0.052	0.052
Activated partial thromboplastin time (s) (n = 559)	0.401(0.322–0.481)	0.015	273.00	0.018	1	0.018
Prothrombin time (s) (n = 562)	0.444(0.378–0.510)	0.166	1.025	1	0.101	0.101
Fibrinogen (g/L) (n = 529)	0.234(0.173–0.295)	< 0.001	1.895	1	0.025	0.025
Procalcitonin (ng/mL) (n = 316)	0.393(0.287–0.499)	0.042	0.1815	0.147	0.872	0.019
Interleukin-6 (pg/ml) (n = 129)	0.253(0.143–0.363)	< 0.001	4.98	0.048	0.963	0.011
Erythrocyte sedimentation rate (mm/h) (n = 326)	0.275(0.201–0.349)	< 0.001	-	-	-	-
CD3^+^ T lymphocytes (/ul) (n = 123)	0.678(0.545–0.811)	0.008	1416.5	0.522	0.82	0.342
CD4^+^ T lymphocytes (/ul) (n = 123)	0.640(0.507–0.773)	0.036	501.0	0.783	0.53	0.313
CD8^+^ T lymphocytes (/ul) (n = 123)	0.671(0.550–0.792)	0.011	421.0	0.696	0.65	0.346

AUC: area under the curve; CI: confidence interval; CD cluster of differentiation.

Statistical differences in the level of Interleukin-6 and procalcitonin among asymptomatic carriers with COVID-19 above the cutoff value compared to those asymptomatic carriers with COVID-19 below the cutoff value were observed (*p* ≤ 0.05; Figure 2);

The area under the curve (*AUC*) was 0.358 for AST (95% *CI*: 0.271–0.445, *p* = 0.004); 0.692 for albumin (95% *CI*: 0.627–0.758, *p* < 0.001); 0.632 for potassium (95% *CI: 0*.545–0.720, *p* = 0.003); 0.648 for sodium (95% *CI*: 0.579–0.718, *p* = 0.001) (Figure 3); 0.401 for APTT (95% *CI*: 0.322–0.481, *p* = 0.015) (Figure 4); 0.234 for fibrinogen (95% *CI*:0.173–0.295, *p* < 0.001) (Figure 4);

More importantly, we obtained the significant diagnostic cutoff value of lymphocytes subsets for distinguishing asymptomatic carriers with COVID-19 with the sensitivity and specificity (Figure 5). Significant differences were detected in the amount of CD3+ lymphocytes, CD4+ lymphocytes, and CD8+ lymphocytes for group 1 (asymptomatic carriers) above the cutoff value in comparison with those below the cutoff value, whose P value were 0.008, 0.036, and 0.011, respectively.

## Discussion

This study’s findings provided epidemiological and clinical data of 648 subjects who comprised 50 asymptomatic carriers, 81 mild cases, 486 ordinary cases, 31 severe or critical cases from 25 hospitals in Jiangsu Province, China. Like the findings from previous studies, fever, cough, sputum production, and fatigue were the main symptoms of COVID-19 [14,15]. The four groups of patients who were categorized according to disease severity, showed significant differences in relation to the leukocyte (*p* < 0.001), lymphocyte (*p* < 0.001), eosinophil (*p* < 0.001), platelet (*p* < 0.001), and neutrophil (*p* = 0.030) levels. Interestingly, we found that the levels of laboratory parameters with liver function, kidney function, and coagulation function were significantly different with disease severity of COVID-19. Also, the levels of inflammatory markers and lymphocyte subsets were characterized with significant differences among them. Additionally, erythrocyte sedimentation rate (ESR) was seen to be significantly associated with disease severity of COVID-19 by multiple regression analysis. The association between ESR as a risk factor and disease severity of COVID-19 had been shown in a statistical analysis. The relationship between ordinary, severe or critical cases and asymptomatic carriers were also observed. The level of lymphocytes, platelet, albumin, fibrinogen, C-reactive protein, CD3 + T lymphocyte, CD4 + T lymphocyte and CD8 + T lymphocyte were independent risk factors for asymptomatic carriers (*p* ≤ 0.05).

SARS-CoV considered as beta-coronaviruses can lead to acute respiratory distress syndrome (ARDS) due to uncontrolled cytokine release such as IL-6 [16,17]. T cells, CD4 + T cells and CD8 + T cells particularly, play a significant antiviral role by balancing the combat against pathogens and the risk of developing autoimmunity or overwhelming inflammation by adaptive immune responses [18,19]. The significant difference in inflammatory markers and lymphocyte subsets (all *p ≤ *0.05) implied that symptomatic patients may have significant immune dysfunction. The novel finding that declined level of serum potassium concentration, sodium concentration, and albumin as well as upward trend of aspartate aminotransferase (AST) were presented among the four groups may be considered to be related to nutritional status and immune response, as Jie Li and Jian-Gao Fan reported in 2020 [20]. Taking these findings together, we conclude that the immune response plays an important role in disease progression, which concurs with the conclusions from similar studies [21]. Therefore, it is critical that the inflammatory mediators generated as part of the immune response are blocked quickly in patients with pneumonia caused by SARS-CoV-2. Moreover, controlling the cytokine storm is vital for patients with severe or critical cases, because this helps to hinder disease progression [22].

Previous study’s findings have indicated that person-to-person transmission can be mediated by patients with asymptomatic COVID-19, and that they should be considered a source of infection [23]. Indeed, the asymptomatic proportion of the novel coronavirus disease (COVID-19) is a useful measurement of the true burden of disease which poses challenges on epidemic prevention and control. This proportion varies widely across infectious diseases, ranging from 8% for measles, 32% for norovirus infections and up to 90–95% for polio [24–26]. Most importantly, for measles and norovirus infections, it is well established that asymptomatic individuals are frequently able to transmit the virus to others [27,28]. Therefore, our study’s ROC analysis determined optimal cutoff values for laboratory variables, including the leukocyte, lymphocyte, eosinophil, platelet, potassium, sodium, AST, ALP, IL-6, CD3+, CD4+, and CD8 + T lymphocyte levels, and the ESR, together with their sensitivities and specificities. These cutoff values could be utilized to identify patients with asymptomatic COVID-19. By helping to identify patients with asymptomatic SARS-CoV-2 infections, these results may help to prevent virus transmission and control the pandemic.

This study has several limitations. The study’s epidemiological and clinical data were obtained from 25 hospitals in Jiangsu Province. These data described the patients’ recent exposure histories, clinical symptoms or signs, laboratory findings, and radiological characteristics on admission to hospital, but some of the data from the 648 subjects were missing or incomplete. Further, our study was not conducted to systematically screen the close contacts of the patients with asymptomatic COVID-19, and it provided no evidence for clusters of infections caused by the transmission of the virus from asymptomatic individuals to apparently healthy people. Since the study’s findings revealed that symptomatic patients may be immunosuppressed, mechanisms underlying patients’ immune responses to SARS-CoV-2 should be explored further.

## Conclusions

We analyzed epidemiological, clinical, and laboratory data from asymptomatic patients and patients who were grouped according to the severity of COVID-19. The groups differed significantly regarding the T lymphocyte response to infection, and our findings suggested that symptomatic patients may experience immunologic disarrangements during disease progression. Considering associations between disease severity of COVID-19 and age, laboratory parameters, as well as ROC analysis for diagnostic predictor of asymptomatic carriers of COVID-19 comprehensively, all these different clinical characteristics should be taken into consideration to identify asymptomatic carriers of COVID-19. Hence, our study’s findings provide novel insights into approaches that may help to prevent virus transmission and control the pandemic.
